# Ultraviolet Weathering Performance of High-Density Polyethylene/Wood-Flour Composites with a Basalt-Fiber-Included Shell

**DOI:** 10.3390/polym10080831

**Published:** 2018-07-27

**Authors:** Van Dinh Nguyen, Jianxiu Hao, Weihong Wang

**Affiliations:** 1 Key Laboratory of Biobased Material Science & Technology (Education Ministry), NortheastForestry University, Harbin 150040, China; dinh77@gmail.com (V.D.N.); axiu518@126.com (J.H.); 2 Vietnam Academy of Forest Sciences, VAFS, Đuc Thang, Bac Tu Liem District, Ha Noi 100000, Vietnam

**Keywords:** wood-plastic composites, ultraviolet radiation, weathering, coextrusion, basalt fiber

## Abstract

In this study, high-density polyethylene (HDPE)/wood-flour composites with a basalt fiber (BF)-reinforced shell were prepared by coextrusion. After exposing these composites to ultraviolet weathering for 2000 h, their performances were examined from their measurements of color, surface morphology, and chemical properties. As a control, UV326 was also added to the shell formula. The weathered surface was characterized by scanning electron microscopy, Fourier transform infrared (FTIR) spectroscopy, and X-ray photoelectron spectroscopy. The results revealed that the shells filled with 8% and 12% BF exhibited low lightness and color change in comparison to those filled with UV326 for a limited duration. The composite shells with the combined BF and UV326 exhibited the least discoloration and surface cracks. FTIR spectra revealed that the oxidation of the composites increases with the duration of exposure to the assessment of the carbonyl group concentration on the surface. The combination of BF and UV326 revealed a synergistic effect on the alleviation of the photooxidation of wood-plastic composite shell layers, verifying the UV-shielding effect.

## 1. Introduction

Wood-plastic composites (WPCs) have been promoted as low-maintenance and highly durable products [1,2]. However, the use of WPCs by the construction industry has led to concerns related to the durability of these products after exposure to outdoor environments. In particular, ultraviolet (UV) durability is of concern because WPCs exposed to accelerated weathering have been reported to exhibit color changes, which affects the aesthetic appeal of the product [3,4,5]. Furthermore, prolonged UV exposure undoubtedly leads to the loss of mechanical integrity [4,6,7,8].

The UV degradation of polymer materials leads to irreversible changes in their chemical structure, which affects the properties of polymers and decreases the useful life of materials. The absorption of ultraviolet (UV) radiation, as well as visible radiation, by colored polymers renders sufficient energy to break several chemical bonds. Such bond breakage is crucial for photodegradation [9,10,11]. Current methods for improving the anti-aging properties of WPCs mainly depend on the use of stabilizers, pigments, and by light-resistant coatings. The manufacturing method, exposure type, and the addition of a photostabilization system have been reported to affect the extent and rate of lightening [5,12,13,14,15,16]. Colorants and ultraviolet absorbers (UVAs) are effective in reducing the fading of wood-flour (WF)/high-density polyethylene (HDPE) composites after accelerated weathering. In addition, the surface chemistry and loss of the mechanical properties of WPC after weathering have been addressed [17,18,19]. Stark and Matuana reported the effects of a UVA, a colorant, and two hindered amine light stabilizers (a low-molecular-weight diester and secondary amine triazine) to the photostabilization of HDPE-based WPC composites by utilizing a factorial design [17]. The addition of a UV stabilizer, such as Tinuvin 783, into HDPE/WF composites revealed that the stability of the HDPE/WF composites to UV treatment depends considerably on the stabilizer content and its dispersion in the polymer matrix [19]. Inorganic pigments, such as titanium dioxide (TiO_2_) and carbon black (CB), have attracted immense attention in recent years because of their ability to absorb UV light and affect the color stability of composites [20,21,22,23,24].

However, the addition of relevant additives into the entire material undoubtedly leads to increased production costs. Coextrusion has been widely utilized in the plastic industry. Two or more extruders are used to simultaneously extrude two or more polymers in a single head and subsequently die in order to produce a multi-layer panel or a sheet structure. This technology has been increasingly used in the development and production of WPCs [24,25,26,27,28,29,30,31]. Coextrusion technology can produce WPCs with different properties for the inner and outer layers. By its functional design of the cap layer (i.e., core shell), the relative performance of WPCs can be improved [31]. In addition to co-extrusion technology, it is important to produce WPCs with age-resistant surfaces.

Basalt fibers (BFs) are relatively new and can be considered to be biological fibers since they are produced from melted natural basalt rocks at a high temperature [32,33]. BFs exhibit the physico-mechanical properties of high-temperature resistance and excellent corrosion, as well as minimal moisture absorption, which is good, sound absorption, and thermal insulation properties [34]. In addition, BFs are cost-effective and high-strength materials that can be used in composites [34,35,36,37]. Chen et al. [38] have reported the effects of adding maleic anhydride grafted high-density polyethylene (MAPE) to basalt fiber, which enhances the mechanical properties of BF-WPCs. Lu et al. [39] have used BFs treated with a coupling agent of vinyl triethoxy silane to improve the mechanical properties of wood-fiber-reinforced HDPE composites. The results revealed improved mechanical properties caused by the increased interfacial compatibility between them. Recently, Wu et al. [40] have used pure HDPE and BF/HDPE layers over a WPC core, which considerably improved the composites impact strength. The flexural and thermal expansion properties wereenhanced using BF-reinforced HDPE shells. Huang et al. [41] have reported similar results for the coextrusion of WPCs with BF and talc filled HDPE shell. However, in the above-mentioned study, the aging resistance of BF-reinforced WPC has not been addressed.

To assist in the development of strategies for decreasing the weathering-induced discoloration of WPCs, BFs were introduced in the cap layer to improve the durability of co-extruded WPC. The uncapped (control) and coextruded WPCs were exposed to accelerated weathering tests, and their surface changes were analyzed by color measurements, scanning electron microscopy (SEM) analysis, Fourier transform infrared (FTIR) spectroscopy, and X-ray photoelectron spectroscopy (XPS). The combination of a UV absorber (UV 326) and BF afforded considerably less resistance in terms of color changes.

## 2. Experimental Section

### 2.1. Materials

High-density polyethylene (HDPE) (5000s grade, melting flow index = 0.8–1.1 g/10 min at 190 °C, density = 0.949–0.953 g/cm^3^) was purchased from Daqing Petrochemical Co., Ltd, Daqing, China. WF from *Acacia mangium* was purchased from Truong Thanh Furniture Corporation, Binh Duong, Vietnam. The moisture content of WF was 18–20%, and WF passed through a 40-mesh screen (the size of each hole in the 40-mesh screen was 0.425 mm). WF comprised a mixture of heatwood and sapwood. BFs (length: 6 mm and diameter: 17 μm) were provided by Zhejiang GBF Basalt Fiber Co., Ltd., Jinhua, China, which were coated with 3-aminopropyltriethoxysilane (KH550) during production to improve the compatibility between BF and polymers. 2-(2-Hydroxy-3′-tert-butyl-5′-methylphenyl)-5-chlorobenzotriazole (UV326), which was used as a UV absorber, was produced by Beijing Specialty Chemicals Co., Beijing, China. MAPE (grafting percentage: 0.9%) was used as a coupling agent and was obtained from Shanghai Sunny New Technology Development Co. Ltd., Shanghai, China. The lubricants (Lub) used were wax and polyethylene (PE) wax.

### 2.2. Sample Preparation

#### 2.2.1. Surface Treatment of BFs

First, a mixed solvent was prepared by mixing 90% anhydrous ethanol and 10% distilled water at room temperature, followed by the addition of KH550. Second, BFs were submerged in the mixed solvent for 30 min and were then filtered from the solution. Next, BF fibers were dried at 120 °C for 2 h before grafting was carried out.

#### 2.2.2. Preparation of Composite Samples

All samples used the same core layer formulation. The shell was divided into four groups: WPC-U; WPC-B; WPC-B-U; and, WPC-B1. Their compositions are listed in Table 1.

WF was dried in an oven at 105 °C to decrease the moisture content to less than 3% (based on dry weight). The dried WF, HDPE matrix, MAPE, BF, UV326, and lubricants were mixed at a specific ratio shown in Table 1. These materials were mixed in a high-speed mixer for 10 min and then fed into a co-rotating twin-screw extruder (SJSH30, Nanjing Rubber and Plastics Machinery Co., Ltd., Nanjing, China) for compounding at 150–175 °C. The extruded, melted mixture, was cooled and reduced into small particles using a pulverizer.

**Figure 1:**
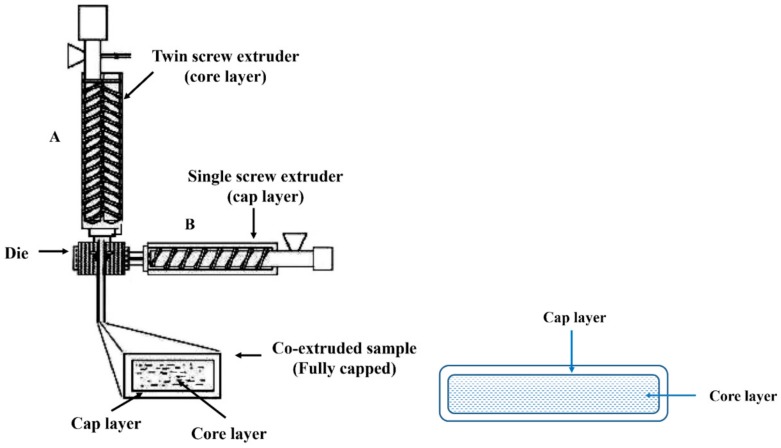
Schematic of the coextrusion system and a cross section diagram of wood flour plastic composites with a core-shell structure [26].

The coextrusion system comprised an SJ45 (A) plastic extruder and an SWMSX-2 (B) plastic extruder (Rubber & Plastics Machinery Co., Ltd., Nanjing, China and SKY WIN Technology Co., Ltd., Nanjing, China) with a screw diameter of 30 mm and an L/d ratio of 36. The pellets of the core raw material were fed into extruder A, and the pellets of the shell material were fed into extruder B (Figure 1). The extruder A barrel temperature was controlled at 155, 160, 165, and 175 °C. The extruder B barrel temperature was controlled at 160, 165, 170, and 175 °C. The samples produced exhibited a cross-sectional dimension of 6 mm (thickness) × 50 mm (width).

The lumbers were cut off and then conditioned at 23 °C and 50–60% relative humidity for one week before testing and weathering treatment.

#### 2.2.3. Accelerated Weathering Treatment

Composite samples were put in an accelerated weathering tester (QUV/Spray, Q-Lab Co., Westlake, OH, USA) and treated according to the procedure of ASTM G154 (Standard Practice for Operating Fluorescent Ultraviolet (UV) Lamp Apparatus for Exposure of Nonmetallic Materials) [42]. Each 12 h weathering cycle consisted of 8 h of UV exposure at 60 °C, followed by 4 h of condensation exposure at 50 °C without irradiation. The UV irradiance was 0.89 W/m^2^ at 340 nm wavelength. The changes in the surface color and morphology of the samples were evaluated after a weathering duration of 0, 500, 1000, 1500, and 2000 h.

### 2.3. Characterization

#### 2.3.1. Colorimetric Analysis

The surface color of the samples were measured using a NF333 photometer (Nippon Denshoku Co., Tokyo, Japan) according to the CIE L* a* b* color system. Lightness (L*) and chromaticity coordinates (a* and b*) were measured for ten replicate samples. An increase in L* signifies that the sample has faded. The total color change (Δ*E*) was calculated using Equation (1) according to ASTM D 2244-02 (Standard Practice for Calculation of Color Tolerances and Color Differences from Instrumentally Measured Color Coordinates) [43],

(1) $$\Delta E=\sqrt{\Delta L^{*2}+\Delta a^{*2}+\Delta b^{*2}}$$

where $\Delta L^{*}$, $\Delta a^{*}$, and $\Delta b^{*}$ are the differences between initial and final values for L*, a*, and b*, respectively.

#### 2.3.2. Scanning Electron Microscopy (SEM)

The surface structure of the co-extruded HDPE/WF composite samples were sputter-coated with gold and analyzed under a scanning electron microscope (QuanTa200, FEI Company, Hillsboro, OR, USA) at the working distance of approximately 25 mm, a voltage of 15 kV, and a probe current of 10 A.

#### 2.3.3. Fourier Transform Infrared (FTIR) Spectroscopy

Surfaces of weathered and unweathered shells were analyzed using an attenuated total reflectance (ATR, MAGNA-IR560, Thermo Nicolet, Madison, WI, USA). Sixty-four scans were recorded with absorbance units from 4000 to 650 cm^−1^. Three spectral measurements were carried out on each sample to study the functional groups on the surface of composites.

#### 2.3.4. X-ray Photoelectron Spectroscopy (XPS) Analysis

An X-ray photoelectron spectroscopy (XPS) analysis was carried out on a K-Alpha spectrograph (Hewlett-Packard Development Company, Palo Alto, CA, USA) using a non-monochromatic Mg source and a take-off angle of 45° relative to the detector. The spot size was roughly 250 μm^2^. A low-resolution scan from 0 to 1100 eV binding energy was used to determine the concentration of each element present on the surface of the samples, along with the oxygen to carbon (O/C) atomic ratio, whereas a high resolution scan from 280 to 300 eV with a take-off angle of 45 °C was performed to further analyze the chemical bonding of the carbon atoms. The binding energy scale was shifted in order to place the main hydrocarbon *C*_1s_ feature at 285.0 eV. To determine the types of oxygen—the carbon bonds present—a chemical bond analysis of carbon was accomplished by curve fitting the *C*_1s_ peak and deconvoluting it into four subpeaks corresponding to an unoxidized carbon, *C*_1_, and various oxidized carbons, *C*_2_, *C*_3_, and *C*_4_. Deconvoluted peak assignments with the corresponding binding energy and bond types are listed in Table 2. An oxygenated to unoxygenated carbon ratio (*C*_ox/unox_) was calculated using Equation (2) [44].

(2) $$C_{ox/unox}=\frac{C_{oxidized}}{C_{unoxidized}}=\frac{C_{2}+C_{3}+C_{4}}{C_{1}}$$

## 3. Results and Discussion

### 3.1. Colorimetric Analysis

**Figure 2:**
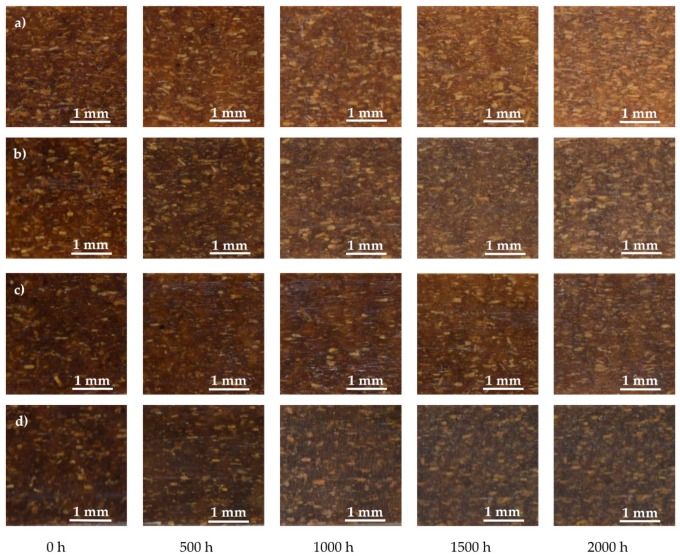
Visual photographs (30×) of all groups of composite shells as a function of the weathering time, with: (**a**) WPC-U comprising 20% WF and 2% UV 326; (**b**) WPC-B comprising 12% WF and 8% BF; (**c**) WPC-B-U comprising 12% WF, 8% BF, and 2% UV 326; and, (**d**) WPC-B1comprising 8% WF and 12% BF.

**Figure 3:**
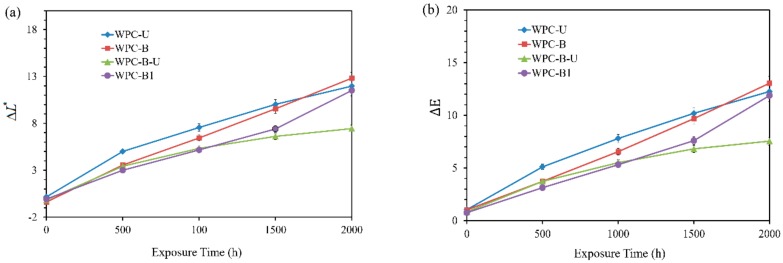
Color parameters, the composites surface at different weathering stages. (**a**) $\Delta L^{*}$, and (**b**) ΔE.

Figure 2 shows the visual appearance of the unweathered and weathered composites after 2000 h. Before aging, the surface color of the samples containing UV326 (Figure 2a) almost retained their original yellow-brown color. On the other hand, for samples with the added BF, their surface color was darker (Figure 2b–d, samples at 0 h). However, as shown in Figure 3, BF did not lead to clear changes in the surface color before aging.

By increasing the aging time, all the samples became lighter and the color changed to a greater extent (Figure 3). $\Delta L^{*}$ was utilized to understand the degree of fading in the weathered HDPE/WF composites, relative to their original color. The most obvious change in lightness was observed in the first 500 h (Figure 3a), indicating that the fabricated composites are distinctly discolored in the first 500 h of weathering. Scissions of the HDPE molecular chain are well known to occur immediately, owing to photodegradation, leading to the decreased density of the HDPE amorphous regions. In addition, the migration of short-chain hydrocarbons to the surface and the photobleaching of lignin lead to the change in lightness [13]. The lightness of WPC-U increased to the greatest extent, while other BF-containing shells changed to a smaller extent, indicating that the BF-containing shell retards lightness to a better extent compared to UV326. This trend was maintained until 1500 h. After 1500 h, the lightness of the shell containing UV326 decreased and became less than that of the shell filled only with BF when the test was carried out at 2000 h. For the shell containing UV326 and BF, it lightened to a marginal extent which was far less than all other formulae, indicating that the simultaneous use of UV326 and BF is better for reducing lightness than using either individually.

Changes in the ΔE values (Figure 3b) for all composites were consistent with those in the $\Delta L^{*}$ values. The ΔE range of the samples, with BF, was 3.7.This value is less than the samples with UV326 (5.11) in the first 500 h of UV weathering. This result revealed that the addition of BF leads to a decrease in the photodegradation rate. By increasing the weathering time, the ΔE change range was similar to that of lightness, which is indicative of the limited long-term photostability of BF. In contrast, the composites with a mixture of BF and UV326 exhibited a minimum Δ*E* value of 7.6 and stabilized, which is indicative of a synergistic effect of BF and UV326. Hence, the ability to resist the effects of UV light and oxidation is enhanced.

At the end of 2000 h of exposure, the total color change and the fade degree of four groups of composites followed the order of WPC-B-U < WPC-B1 < WPC-U < WPC-B. Based on these test results, a mixture of BF and UV326 can be suitable for applications that require color stability for improving WPC color performance. In addition, for a short-life duration, BF can provide better protection for the shell in comparison to UV 326.

### 3.2. Surface Morphology of Composites

**Figure 4:**
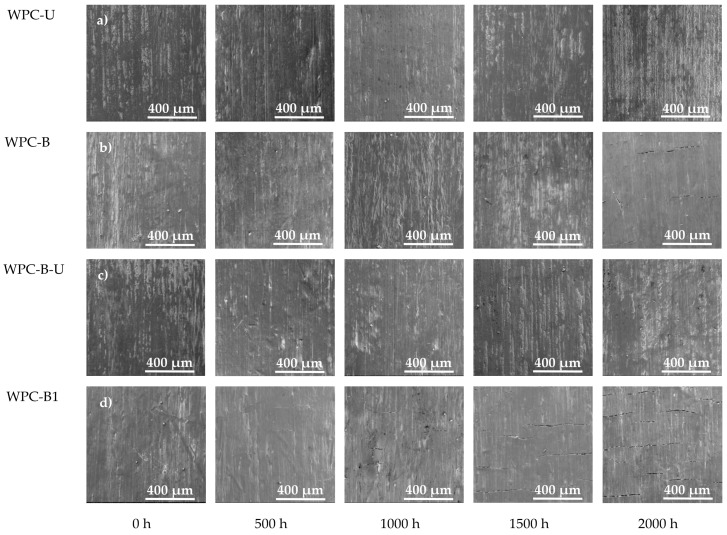
Scanning electron microscopy (SEM) micrographs (100×) of composites at different times after accelerated UV weathering for 0, 500, 1000, 1500, and 2000 h.

In addition, the surface morphology of the composites significantly changed after weathering. The SEM micrographs of the shell layer of WPC composites revealed the coarsening of the initially smooth surfaces (Figure 4) after accelerated UV weathering. This effect was visible from 1000 h for the sample containing 12% BF and 2000 h for the 8% BF sample. Coarsening was accompanied by matrix cracks. After 2000 h of weathering, the composite surface with the added 12% of BF was the most severely degraded (Figure 4d), while the sample surfaces prepared using the UV326 stabilizer revealed partial changes in the surface and not degradation (Figure 4a,c). This result might be mainly related to the thermal effect. Thermal expansion was different inthe HDPE matrix and the BF (HPDE values from 15 to 30 × 10^−5^/K and BF values from 279.7 to 281.2 × 10^−6^/K) [45], which causes stress at the interface between the 50 and 60 °C testing environment. The shell, by the addition of 8% BF, was less affected and afforded smaller cracks (Figure 4b). With the progress of UV treatment, the protrusion of BF and wood particles from the HDPE matrix was observed because of its degradation.

On the other hand, UV-326 powder was enveloped in the HDPE matrix. Upon exposure to UV weathering, the particles tended to migrate to the surface, which protected HDPE from degradation via UV absorption. This absorption possibly decreased the accumulated thermal effect from UV and restrained the detachment of BF from HDPE. At the end of 2000 h of weathering, the composite shell containing BF and UV326 exhibited marginal effects from UV aging; the least amount of change was in lightness, color, and surface morphology. BF and UV326 possibly exhibited a synergistic effect, leading to the enhancement of UV-induced oxidation resistance.

### 3.3. Surface Chemistry Analysis by FTIR

**Figure 5:**
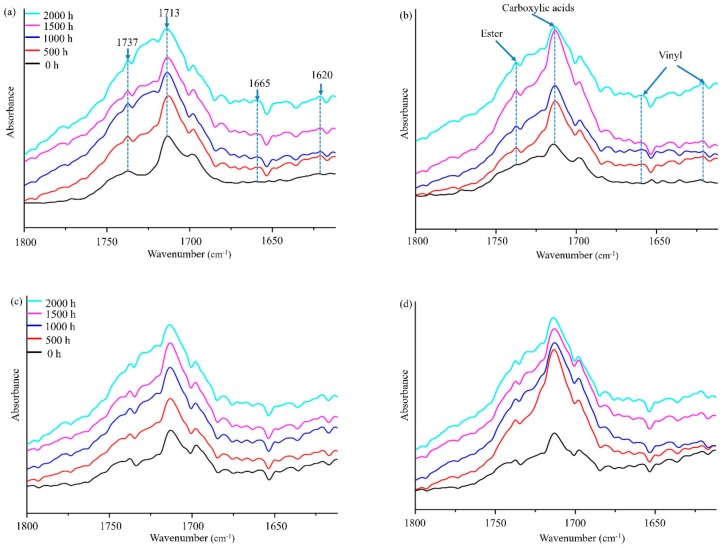
FTIR spectra of coextruded WPC composites before and after weathering: (**a**) WPC-U;(**b**) WPC-B; (**c**) WPC-B-U; and, (**d**) WPC-B1.

FTIR spectra revealed changes in the surface chemistry of the composites during UV-accelerated weathering (Figure 5). Characteristic peaks of polyethylene were observed at 1737, 1713, and 1670–1610 cm^−1^. Table 3 summarizes the assignments for the corresponding functional groups.

In the carbonyl region (1750 to 1710 cm^−1^), a sharp peak was observed at 1713 and 1737 cm^−1^, corresponding to the hydrogen-bonded carboxylic acid and the ester groups, respectively [46]. Polyolefin is well known to mainly undergo two chain fracture reactions, Norrish I and Norrish II, under UV irradiation, affording a carbonyl group and a vinyl group, respectively. The short chain undergoes a cross-linking reaction [47]. In the Norrish I reaction, the resulting free radicals can attack the polyolefin, which may lead to chain scission or crosslinking. In the Norrish II reaction, vinyl groups and terminal carbonyl groups are produced and a chain scission occurs [7,48,49]. By increasing the exposure time, the content of the carbonyl and ester groups increased at approximately the same rate for all composites. The increase in the carbonyl and ester group formation for composites after weathering is known to be proportional to the number of chain scissions occurring in HDPE. After 1000 h of exposure, the band corresponding to the carbonyl groups stopped increasing with the exposure time, which is possibly related to the physical wash-off of the oxidized materials at the sample surface—further confirming the hypothesis made from color changes (Figure 3).

The peak was located at 1731 cm^−1^, corresponding to C=O stretching, which clearly increased after weathering the BF sample for 1000 h, indicating that surface oxidation occurs in the first 1000 h of exposure. The carbonyl group index increased, which is the main result obtained from the thermo and photooxidation of polyethylene. The sample with 8% BF and 12% BF revealed higher-carbonyl-group absorbance (Figure 5b,c)—i.e., a higher degree compared to the sample prepared with added UV326.

The sample with the added UV326, and a mixture of BF and UV326, revealed lower carbonyl group absorbance (Figure 5a,c), i.e., a lower degradation degree compared to the sample with the added BF (Figure 5b,d), related to the prominent light-absorption capability of UV326. When compounding UV326 and BF (Figure 5c) as the anti-UV agent, the surface chemical bonds exhibited few changes after 1000 h of UV-accelerated weathering; the duration for resistance to UV-accelerated weathering was prolonged up to 2000 h based on the above analysis of color and surface morphology in this study. This indicates that the addition of this compound system exhibits excellent durability and good application prospects.

The absorption peaks of the vinyl groups ranging from 1670 to 1610 cm^−1^ were stable at 1000 h for WPCs with the added UV326 and BF (Figure 5), and did not change after 2000 h of weathering.

### 3.4. XPS Analysis

Table 4 summarizes the XPS results obtained from the surface elemental analysis of the shell surface before and after UV weathering.

Table 4 summarizes the atomic concentrations of oxidized carbons, i.e., carbon atoms connected to oxygen atoms (*C*_2_, *C*_3_, and *C*_4_) and unoxidized carbon (*C*_1_) obtained from the assignment. The deconvolution of the carbon peak revealed that the relative amount of *C*_1_ carbon, representing a C–C or C–H bond, decreased after weathering by the addition of UV326 and increased by the addition of BF in the composites (Table 4). XPS data listed in Table 4 also reveals an increase in the total oxygenated carbon bonds by the addition of UV326 and decreased with the addition of BF, *C*_ox/unox_, suggesting that surface oxidation occurs. This result implied that significant surface oxidation occurs after 2000 h of exposure. The change in the total oxygenated carbon bonds may be attributed to the degradation of the matrix.

Some researchers have revealed that the surface elements of BF can form hydrogen bonds with hydrophilic polar groups and the BF surface contains a high Si content, which demonstrates a potential to react chemically with the surrounding, active functional groups, under certain conditions [50,51]. Furthermore, in addition to the increased compatibility of the BF and the WPC, a closer proximity between the groups that participate, in these hydrogen bonds or chemical reactions and the WPCs, can facilitate hydrogen-bond formation or chemical reactions [52]. These results suggested that by increasing the weathering duration, there was an increase in the small cracks observed on the sample surface, of the 8% BF and 12% BF samples, which was consistent with the SEM results.

## 4. Conclusions

In this study, the effect of accelerated weathering on the surface properties of polyethylene composites reinforced with BF and UV326 at different loading levels was investigated. Based on the analysis, the following conclusions were made:

The UV weathering resistance of the composites was considerably effected by the type of additional reinforcement used. FTIR analysis demonstrated that significant changes to the surface chemistry of the composite material, after exposure, are observable. After 1000 h, the characteristic peaks of C=O (i.e., carbonyl and ester groups) clearly increased. The WPC composites, with the combined BF and UV326, exhibited the highest stability during the entire weathering duration.

The combination of BF and UV326 exhibited a synergistic effect on the retardation of the photo-oxidative aging of the HDPE/UV326 shell layers, which confirmed their UV-shielding effect. These results provided a theoretical reference for the development of WPC composites for outdoor use, which have better photostability.

## Figures and Tables

**Table 1:** Formulations and materials used in the core and shell layers of HDPE/WF composites (wt %).

Layer	WF	HDPE	MAPE	Lub	UV326	Basalt fiber	Sample code
Core	60	37	3	2 ^a^			
Shell	20	77	3	2 ^a^	2 ^a^		WPC-U
	12	77	3	2 ^a^		8	WPC-B
	12	77	3	2 ^a^	2 ^a^	8	WPC-B-U
	8	77	3	2 ^a^		12	WPC-B1

^a^: The weight percent of UV326 and Lub was based on the total amount of WF, HDPE, MAPE, and BF.

**Table 2:** Deconvoluted peak assignments with corresponding theoretical binding energy and bond type for high-resolution XPS scans of *C*_1s._

Carbon Group	Binding Energy (eV)	Bond
*C*_1_	285.0	C–C or C–H
*C*_2_	286.5	C–O
*C*_3_	288.0	O–C–O or C=O
*C*_4_	289.5	O–C=O

**Table 3:** Assignment of IR spectra absorption bands.

Wavenumber (cm^−1^)	Assignments [46,47]
1737	C=O stretching (ester carbonyl)
1713	C=O stretching (acid carbonyl)
1670–1610	C=C stretching (vinyl groups)

**Table 4:** Relative amount of different carbon-to-oxygen bonds at the sample surface determined by high-resolution XPS.

Sample	Analysis of *C*_1s_ Peaks (%)				
	C_1_	C_2_	C_3_	C_4_	*C*_ox/unox_
Sample with UV326					
Unweathered	76.05	13.77	8.37	1.81	0.31
Weathered for 2000 h	73.37	18.07	5.78	2.77	0.36
Sample with BFs					
Unweathered	78.30	12.30	7.73	1.67	0.28
Weathered for 2000 h	81.01	9.67	5.14	4.18	0.23
Sample with UV326 and BF					
Unweathered	80.04	9.89	8.11	1.96	0.25
Weathered for 2000 h	81.16	10.71	4.54	3.58	0.23
Sample with BF1					
Unweathered	77.97	12.58	7.77	1.68	0.28
Weathered for 2000 h	82.11	10.28	4.56	3.04	0.22
